# Sexual Harassment in the Hospital: Insights from Medical residents and interns in Sfax Tunisia

**DOI:** 10.1192/j.eurpsy.2025.2289

**Published:** 2025-08-26

**Authors:** S. Ajmi, F. Cherif, O. Bouattour, D. Mnif, I. Feki, R. Masmoudi, J. Masmoudi

**Affiliations:** 1 Psychiatry A department, Hedi Chaker hospital university, Sfax, Tunisia

## Abstract

**Introduction:**

Sexual harassment (SH) is defined as any form of pressure aimed at obtaining a sexual act, for the benefit of the perpetrator or a third party. Despite movements aimed at combating sexual harassment, it remains a complex and concerning reality, particularly in the workplace. Unfortunately, the hospital environment is not spared from this issue. Medical residents and interns seem to be the population most affected by the SH.

**Objectives:**

To estimate the prevalence of SH among medical residents and interns.

To describe the profile of SH’s victims and examine their perceptions regarding sexual education.

**Methods:**

We conducted a cross-sectional and descriptive study involving medical residents and interns working in hospitals in Sfax. Data were collected using an anonymous self-questionnaire. This questionnaire was published on social media during January and February 2024. It included sociodemographic characteristics, psychiatric and medical history, psychoactive substance use, professional data, information related to sexual life and experiences related SH.

**Results:**

We received 141 responses, of which 19.9% declined to participate in this study. Ultimately, 113 participants, with an average age of 27.92 years, were included in the final analysis. The sex ratio (M/F) was equal to 0.54. In our population, 20.4% were interns. Among the participants, 68.1% were single, 91.2% were from urban origine and 12.4% had psychiatric follow history for anxiety disorders, depression, obsessive-compulsive disorder, and bipolar disorder (n= 8, 2, 2, and 1, respectively).

Among the participants, 41.6% reported experiencing sexual harassment during their practice at the hospitals in Sfax. The most common form reported as harassment was verbal harassment (43,3%). The majority of SH perpetrators were male (87.2%), and 27.6% were senior physicians. According to our study, 8.5% of the SH perpetrators were sanctioned for their actions, and 80.5% of SH victims believed that the perpetrators had continued to exhibit such behavior.

Regarding sexual education, 80.5% of participants thought that it was necessary in school curriculums, with thirty-seven participants believing they had received adequate sexual education.

**Conclusions:**

This study highlighted the high prevalence of sexual harassment among medical trainees in Sfax. These results serve as an urgent call to the development of effective strategies to prevent sexual harassment in hospital environments and highlight the importance of raising awareness among staff about available legal protections.

**Disclosure of Interest:**

None Declared

